# From the Editors

**DOI:** 10.35946/arcr.v41.1.10

**Published:** 2021-07-29

**Authors:** John F. Kelly, Brett Hagman

**Affiliations:** 1 Department of Psychiatry, Massachusetts General Hospital, Recovery Research Institute, Boston, Massachusetts; 2 Department of Psychiatry, Harvard Medical School, Boston, Massachusetts; 3 Division of Treatment and Recovery Research, National Institute on Alcohol Abuse and Alcoholism, National Institutes of Health, Bethesda, Maryland

In recent decades, the term “recovery” as it pertains to alcohol use disorder (AUD) and drug use disorders has taken on increasing cultural and scientific significance in the United States and around the world. Its growing prominence as a culturally recognized and, importantly, positively valenced organizing concept has occurred in large part in direct response to help counter the pervasive and intransigent stigma, discrimination, and general pessimism that so often surround alcohol misuse and AUD.^1^–^3^ In addition, the term “recovery” often is used intentionally to describe improvements in functioning and quality of life that go beyond solely abstinence or disorder remission.^4^,^5^ This broader construct stands in explicit contradistinction to the mere absence of alcohol use or AUD symptoms. As the cultural significance of recovery has developed and deepened, the scientific community has become interested in understanding its meaning, both as a dynamic, multidimensional biobehavioral process and as an outcome. Moreover, given the burden of disease, disability, mortality, and economic costs attributable to AUD, the discovery of factors that can help affected individuals to initiate and sustain long-term stable AUD recovery has become paramount. With all of these ends in mind, this topic series, “Recovery From Alcohol Use Disorder,” reviews current understanding of AUD recovery from clinical, public health, and public policy perspectives.

Drawing on the expertise of renowned AUD researchers, this series provides an expansive review of what is currently known about recovery from AUD. From defining what “recovery” is to describing its epidemiology; its salubrious neurological, somatic, psychological, and behavioral effects; the services and therapeutic factors responsible for helping individuals initiate and sustain it; and the myriad pathways followed to achieve it—this series covers expansive terrain.

Defining what recovery actually is has been a goal of many organizations and stakeholder groups in recent years, including the National Institute on Alcohol Abuse and Alcoholism (NIAAA). This series begins with an in-depth look at defining recovery, examining the nuances and presumed components of the domain with important implications for clinical research and public health (Witkiewitz, Montes, Schwebel, et al., 2020).^6^ Recovery prevalence also has been of great interest, including the extent to which individuals self-identify as “a person in recovery” (or not) and which demographic and clinical subgroups of individuals appear to have fewer or greater challenges on the path to recovery than others. Some of the reasons for these differences are detailed and explained along with the known estimates of recovery prevalence in the United States (Tucker, Chandler, and Witkiewitz, 2020).^7^ The positive neurophysiological, somatic, psychological, and behavioral effects of, and the milestones involved in, AUD recovery are of great interest to affected parties, as well as to the public and the clinical and research fields. These effects are covered in detail across domains of brain (Nixon and Lewis, 2020)^8^ and other organ systems (Thomes, Rasineni, Saraswathi, et al., 2021).^9^

Several articles describe the therapeutic and dynamic mobilizers of recovery-related change across various clinical, nonclinical, and self-management pathways, including articles about the recovery journey (Davidson, Rowe, DiLeo, et al., 2021; Stout, 2021)^11^,^12^ among individuals and their families (McCrady and Flanagan, 2021).^10^ This section includes articles on long-term clinical in-person care (McKay, 2021),^13^ pharmacology (Mason and Heyser, 2021),^14^ and the growing array of community-based recovery support services, such as mutual help organizations (Zemore, Gilbert, Pinedo, et al., 2021),^15^ as well as recovery housing, recovery coaching, recovery supports in education, and recovery community centers (Jason, Salomon-Amend, Guerrero, et al., 2021).^16^ Demographic and clinical factors that have been shown to affect initiation and trajectories of recovery and related change are featured in depth with specific focus on sex (Holzhauer, Cucciare, and Epstein, 2020),^17^ age (Finch, Jurinsky, and Anderson, 2020),^18^ and race and ethnicity (Wagner and Baldwin, 2020).^19^

In sum, during the past 50 years since the birth of NIAAA, and strongly influenced by the voluminous research it has generated, the field has witnessed a number of evolutionary paradigm shifts in understanding and approach that have informed how best to address the endemic problems associated with alcohol misuse and AUD. This landmark topic series reflects yet another shift—one that recognizes the necessity of attending not only to clinical pathology through acute stabilization and short-term, professionally delivered services, but also to the need for additional resources to help individuals and their families build resilient, robust recovery and permit human flourishing over the long term.
